# Treatment of Sarcoidosis: A Multidisciplinary Approach

**DOI:** 10.3389/fimmu.2020.545413

**Published:** 2020-11-19

**Authors:** Alicia K. Gerke

**Affiliations:** Pulmonary and Critical Care Medicine, Department of Internal Medicine, University of Iowa, Iowa City, IA, United States

**Keywords:** therapeutics, immunosuppression, sarcoidosis, drug-related side effects and adverse reactions, treatment outcome, granuloma

## Abstract

Sarcoidosis is a systemic disease of unknown etiology defined by the presence of noncaseating granulomatous inflammation that can cause organ damage and diminished quality of life. Treatment is indicated to protect organ function and decrease symptomatic burden. Current treatment options focus on interruption of granuloma formation and propagation. Clinical trials guiding evidence for treatment are lacking due to the rarity of disease, heterogeneous clinical course, and lack of prognostic biomarkers, all of which contribute to difficulty in clinical trial design and implementation. In this review, a multidisciplinary treatment approach is summarized, addressing immunuosuppressive drugs, managing complications of chronic granulomatous inflammation, and assessing treatment toxicity. Discovery of new therapies will depend on research into pathogenesis of antigen presentation and granulomatous inflammation. Future treatment approaches may also include personalized decisions based on pharmacogenomics and sarcoidosis phenotype, as well as patient-centered approaches to manage immunosuppression, symptom control, and treatment of comorbid conditions.

## Introduction

Sarcoidosis is a systemic disease of unknown etiology that can cause organ dysfunction and diminished quality of life. The disease is diagnosed by a constellation of radiographic, clinical and histopathologic findings; it is most often defined by the presence of noncaseating granulomatous inflammation that occurs in the absence of infection, exposures, malignancy, or alternative immune-related disease. Lung and thoracic lymph nodes are most often involved, but any organ can be affected, with multi-system involvement having a worse prognosis. Treatment is directed at alleviating organ dysfunction, preventing irreversible scarring, and improving quality of life. Herein, we review the indications for treatment, pharmacotherapy, treatment duration, side effects, adjunct non-pharmacologic therapies, and outcomes for patients.

## Brief Pathogenesis

The sarcoidosis granuloma is formed by a distinct conglomeration of multinucleated giant cells and epithelioid macrophages surrounded by a rim of CD4+ T cells (1). Less abundantly, CD8+ T cells and B cells can be found in the surrounding rim. The granulomatous inflammation seen in sarcoidosis is thought to be a dysregulated antigenic response due to an unknown environmental exposure in a genetically susceptible individual. Loci that house antigen presentation genes such as HLA class II and BTNL-2 have been linked to development of sarcoidosis, as well as certain disease phenotypes (2–4). Mycobacterial antigens have been proposed based on studies showing heightened immune responses of both peripheral macrophages and bronchoalveolar (BAL) fluid of patients with sarcoidosis to mycobacterial proteins including mKatG and ESAT-6 (5, 6). Similarly, *Propionibacterium acnes* has also been proposed as an etiologic agent given the higher frequency of genetic material found in sarcoidosis granulomas compared to controls, as well as similar exaggerated immune response to *Priopionibacteria* in sarcoidosis T cells compared to normals (7). It is also likely that dendritic cells play an important role in the presentation of antigen and continued immune response, although the mechanisms are not well-understood (8). More recent data would also suggest that not only is sarcoidosis a disease of heightened Th1 immune response, but also potential dysfunction of regulatory immune cells and immune ‘exhaustion’ with failure to clear an antigenic agent (9, 10). Therefore, therapeutic targets currently include suppression of inflammation, improvement of the regulatory capacity of the immune system, and modulating the antigen or antigen presenting capacity of the immune system.

**Figure 1:**
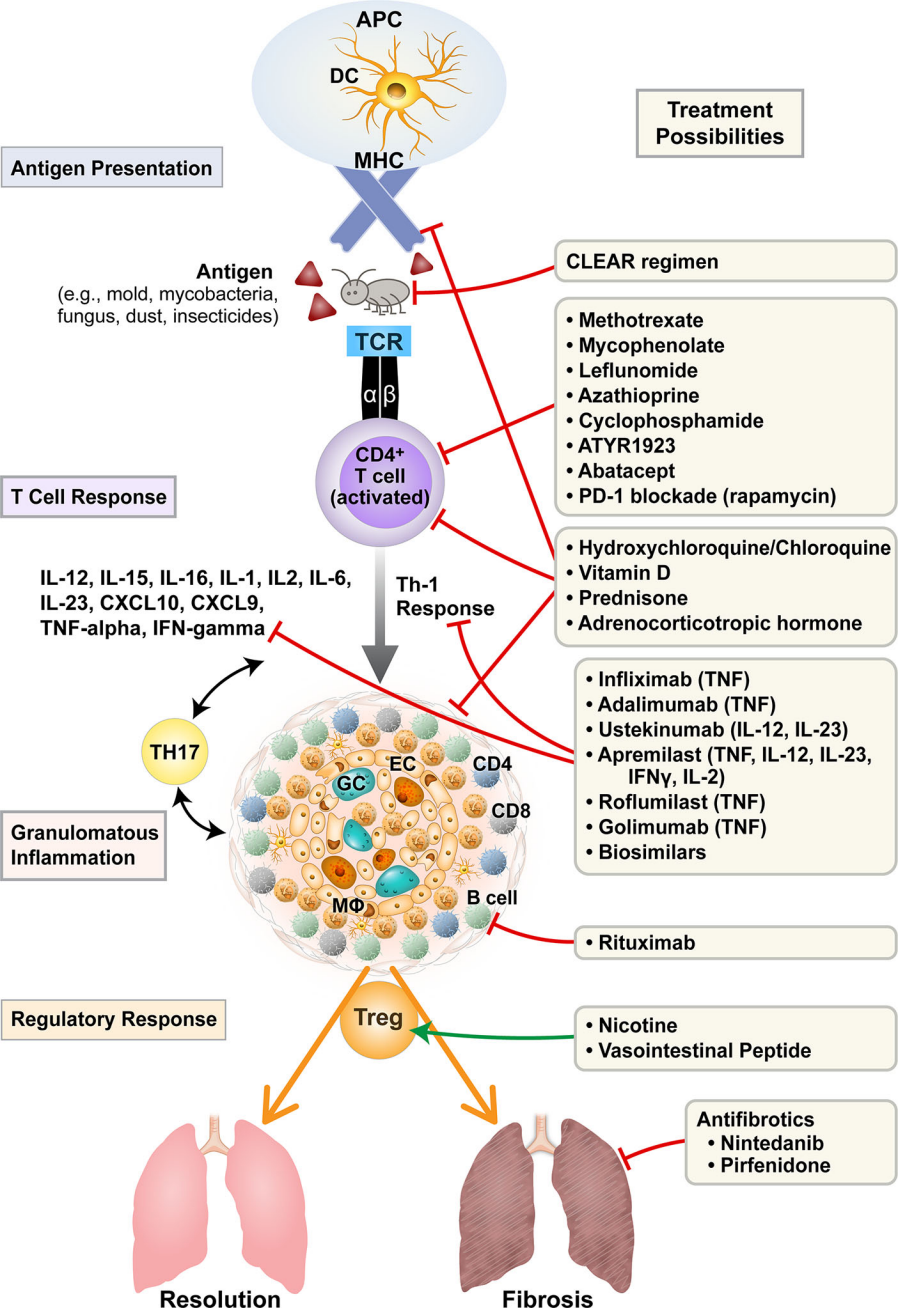
Current and investigational treatments for sarcoidosis based on pathogenesis. Treatments for sarcoidosis target antigen presentation, T cell activation, cytokine/chemokine profiles, propagation of granulomatous inflammation, T-regulatory balance, and the fibrotic response. APC, antigen presenting cell; DC, dendritic cell; MHC, major histocompatibility complex; TCR, T cell receptor; GC, multinucleated giant cell; EC, epitheloid cell; Mφ, macrophage; IL, interleukin; TNF, tumor necrosis factor; IFN, interferon; PD-1, programmed cell death protein-1; CLEAR, Combined Levofloxacin; Ethambutol, Azithromycin and Rifampin.

The basis of treatment of sarcoidosis is regulation of the heightened immune response and suppression of granulomatous inflammation in order to prevent dangerous interference with organ function (as seen in the eye or the heart) and to prevent eventual scarring and fibrosis as seen in the lungs. Current standard of care focuses on suppressing highly activated macrophages and T cells and their production of cytokines such as TNF-α, IL-1, IFN-γ, and IL-6. More recent study has also suggested a possible role of anti-B cell therapy given the presence of B cells in the granuloma and increased B cell activating factor (BAFF) in sarcoidosis patients (11). Additionally, the increasing evidence of a Th17 response (as seen in autoimmune disease) in addition to a strong Th1 mediated response potentially suggests more therapeutic targets (12). Last, cytokine-specific biologics and treatment of mycobacterial infection have emerged as potential future alternatives. **Figure 1** illustrates current and investigational therapies for sarcoidosis based upon pathogenesis.

## Treatment Approach

**Figure 2:**
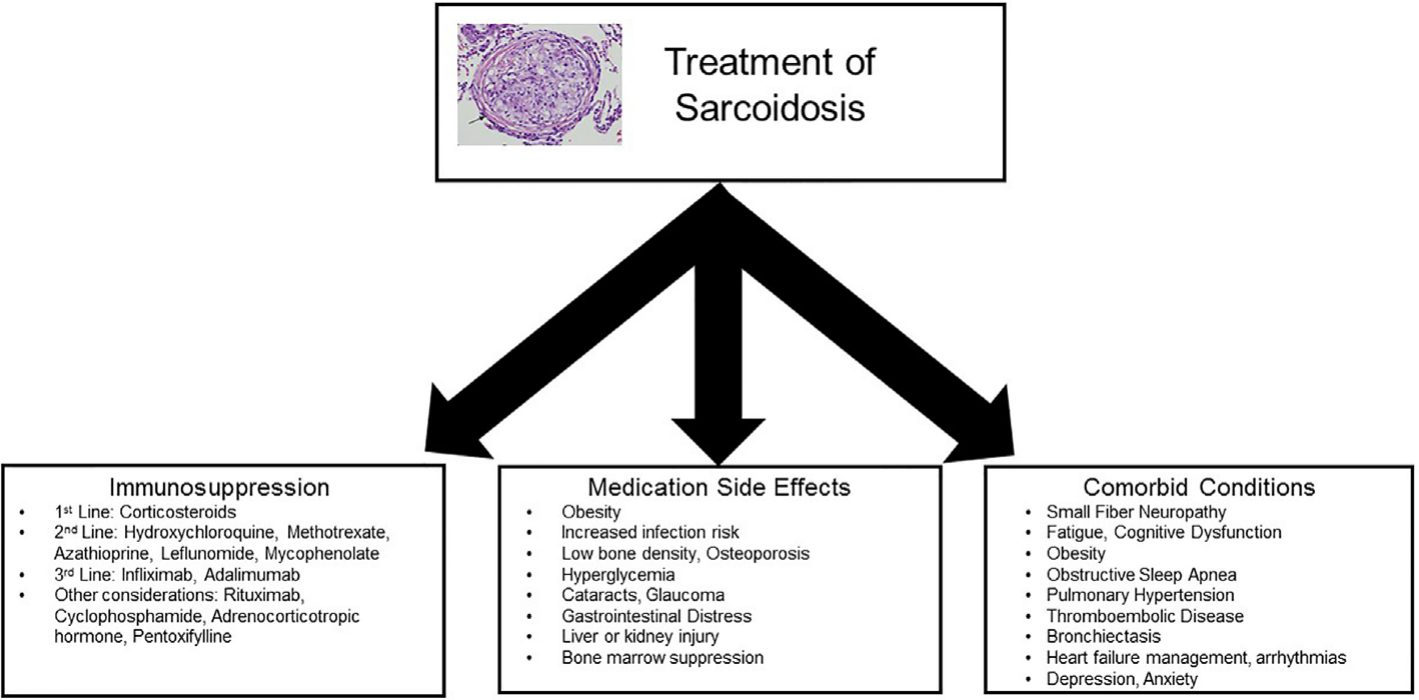
Concepts for treatment of sarcoidosis. Management of patients with sarcoidosis often requires a multidisciplinary approach: treatment of symptomatic granulomatous inflammation, assessment of comorbid conditions, and tempering of immunosuppressive toxicities.

To date, treatment of sarcoidosis is largely guided by small, uncontrolled trials and expert consensus (13–19). A few randomized controlled trials (RCTs) have been performed, but trials are limited by the rarity of disease, heterogeneity of disease presentation and progression, and lack of standardized, responsive outcome measures (20, 21). Additionally, even past negative trials may not completely negate the efficacy of certain compounds, as study design is difficult due to the idiosyncrasy of the disease itself (21). A number of suggested treatment algorithms have been published, most of which are based upon initiation of corticosteroids in a symptomatic patient with abnormal function or imaging studies, followed by tapering of steroids over a minimum of 1 year (22, 23). Second and third-line agents can be added based on lack of response to therapy, toxic side effects, or inability to taper corticosteroids (**Table 1**). Importantly, outcomes of symptomatic response and toxicity profile can vary from patient to patient, emphasizing the need for a patient-centered approach. Objective outcomes can include imaging studies (e.g., chest X-rays, computed tomography (CT), positron emission tomography (PET) scans, MRI), pulmonary function tests, walk distance, and laboratory testing (e.g., liver function, blood counts, chemistries, calcium). Subjective assessments can include (but are not limited to) dyspnea, cough, fatigue, cardiac symptoms, neurologic symptoms, and pain. In some patients, the objective and subjective outcomes may not correlate, adding to the complexity of management decisions (24). Therefore, management of patients with sarcoidosis often requires a three-pronged approach: treatment of symptomatic granulomatous inflammation, assessment of comorbid conditions, and tempering of immunosuppressive toxicities (**Figure 2**).

**Table 1:** Common therapeutics for treatment of sarcoidosis*.

	Drug name	Suggested dose range	Special treatment issues/monitoring
**First-Line Agents**	Corticosteroids (Prednisone)	20–40 mg/day initial Dose, tapered to 7.5–15 mg/day	Bone density Eye exams (glaucoma and cataracts) Body Mass Index
**Second-Line Agents**	Methotrexate	7.5–25 mg/week orally or subcutaneously	Concurrent need for folic acid. Liver function, kidney function, CBC. Can cause hepatotoxicity, GI distress, pneumonitis, mouth ulcers, bone marrow suppression.
	Hydroxychloroquine	200–400 mg/day	Eye exams for retinopathy. Rarely associated with QT elongation (consider drug interactions).
	Leflunomide	10–20 mg/day	Liver function, kidney function, CBC. Can cause neuropathy, hepatotoxicity, GI distress, pneumonitis, bone marrow suppression. In cases of toxicity, can clear more urgently with cholestyramine.
	Azathioprine	50–200 mg/day	Liver function, kidney function, CBC. Consider TPMT level. Can cause hepatotoxicity, GI distress, hypersensitivity reaction, bone marrow suppression.
	Mycophenolate	500–3,000 mg/day	Liver function, kidney function, CBC. Associated with GI distress, bone marrow suppression. Enteric coated option available (different dose range).
**Third-Line Agents**	Infliximab	3–5 mg/kg intravenously at weeks 0, 2, and every 4–8 weeks thereafter	Tuberculosis Testing Caution in heart failure. Allergic reactions possible with injections. Associated with demyelination syndrome, malignancy, and sarcoid-like reactions.
	Adalimumab	40 mg subcutaneous every 1–2 weeks	Similar precautions and adverse reactions as infliximab.

Other therapeutic options can be considered in some cases (e.g., cyclophosphamide, rituximab, adrenocorticotropic hormone, pentoxifylline).

CBC, complete blood count; GI, gastrointestinal; mg, milligrams; kg, kilograms; TPMT, thiopurine methyltransferase.

## Indications for Treatment

Over half of patients with sarcoidosis will incur spontaneous resolution or never have clinical manifestations of the disease, whereas the remaining half will experience a more chronic course, often requiring treatment. Mortality appears to be increasing over time, and burden of disease can be substantial due to complications of the disease or its treatments (25). It is unclear if treatment with corticosteroids alters the natural history of disease; therefore, treatment is recommended only for those with high symptom burden and/or evidence of organ damage (26). For those requiring treatment due to symptoms or suspicion of injury to organs, a stepwise approach to therapy is recommended, although more aggressive therapeutic management can be considered in particularly severe cases of neurologic, ophthalmic, or cardiac involvement (23). In general, there are very few large clinical trials for treatment in sarcoidosis (27). Therefore, in clinical practice, use of corticosteroids and other immunomodulatory agents is often modeled by use on other autoimmune and inflammatory diseases in which suppression of the immune system is desired.

## Immunosuppressive Treatment

### Corticosteroids

Corticosteroids are considered first-line treatment by consensus of sarcoidosis providers (28, 29). Multiple uncontrolled studies have shown that corticosteroids suppress production of cytokines that contribute to persistent granuloma formation including TNF-α and IFN-γ (30, 31). However, only a small number of RCTs with corticosteroids have been performed (26). Although patients have shown improvement in symptoms and biomarkers in the short-term, current evidence is yet unable to show long-term benefit, alteration of natural history or improved mortality (32). Accordingly, the ideal dosing and length of therapy are unknown. A starting dosage of 20–40 milligrams (mg) per day is generally recommended, although a few select patients with severe disease may be initiated on much higher doses in cases of severe neurosarcoidosis, refractory arrhythmias, ophthalmic disease threatening vision loss, or other severe organ damage (29). Lower induction doses may also be reasonable, particularly in pulmonary sarcoidosis, as one early clinical trial of prednisone established improvement in pulmonary infiltrates with 15 mg per day (33). Similarly, a Finnish study from 2002 in pulmonary sarcoidosis used a protocol of 20 mg of prednisolone per day for eight weeks, 15 mg per day for 2 weeks, 10 mg per day for 2 weeks, and then inhaled budesonide for 15 months. The results showed improved pulmonary function over a five-year period in patients with Stage II-III disease (34). Long-term doses above 40 mg per day are not recommended for most cases of sarcoidosis due to high risk of corticosteroid-induced toxicity and little added physiologic benefit. For example, a study in Japan revealed increased morbidity and mortality in patients with cardiac sarcoidosis who were treated with higher doses of corticosteroids (greater than 40 mg per day) compared to lower doses (less than 30 mg per day) (35). Similarly, higher cumulative doses of prednisone in sarcoidosis have been associated with decreased quality of life and increased frequency of emergency department visits (36). Another analysis of differing starting dosages for patients with pulmonary sarcoidosis did not show a strong correlation between outcome and initial dose, indicating that lower doses may be used with similar effect. On the other hand, there was a strong correlation between starting dose and weight gain over a two-year period (37). Taken in total, these studies suggest that toxicity of higher dose corticosteroids is prominent, whereas there appears to be no discernible benefits in disease outcomes on higher doses versus lower doses, particularly for maintenance therapy.

### Corticosteroid-Sparing Medications

Although corticosteroids are most often used first-line for sarcoidosis, patients with chronic sarcoidosis requiring prolonged therapy, those with particularly severe disease, and those with significant corticosteroid toxicity may require a corticosteroid-sparing medication. A Delphi consensus of sarcoidosis experts noted that doses of greater than 10 mg of prednisone per day were generally considered too high for long-term therapy and a steroid-sparing agent is often considered in these patients (29). Methotrexate, azathioprine, leflunomide, and mycophenolate are the most common steroid-sparing alternatives applied to treat sarcoidosis. Methotrexate is the most frequently recommended second-line therapy, based on its well-established side effect profile and efficacy in autoimmune disease such as rheumatoid arthritis and psoriasis (29). It is a folic acid antagonist that, through a series of steps, inhibits purine and pyrimidine metabolism, as well as amino acid and polyamine synthesis. The drug may also have other anti-inflammatory effects on T cells, suppressing formation of contributing cytokines by these cells *via* the adenosine a2a receptors (38). Methotrexate has been shown to be an effective steroid-sparing agent in two small clinical intervention studies (one randomized and one non-randomized), where patients showed improvement in vital capacity or symptomatic organ dysfunction while concurrently tapering down on steroids (39, 40). These data are supported by multiple case series and retrospective studies, both in pulmonary and extrapulmonary sarcoidosis (19). A dosage of 7.5 mg to 15 mg per week appears to be effective for most cases, with an overall response rate up to 55%, which may be higher if used in combination with corticosteroids and lower if used as monotherapy (19, 41). However, a significant percentage of patients both in sarcoidosis and rheumatoid arthritis do not respond or have toxicity to methotrexate, which may be reflective of certain pharmacogenomic profiles or genetic predisposition to drug efficacy and/or toxicity (42, 43). The most common adverse events seen with treatment with methotrexate include infections, hepatotoxicity, gastrointestinal distress, malaise, and leukopenia. Patients should be started on folic acid concurrently with methotrexate and regular monitoring of liver function, blood counts, and kidney function should be performed.

Similarly, leflunomide, a dihydroorotase inhibitor that inhibits dividing lymphocytes, is often used an alternative (or even additionally, in some cases) to methotrexate in sarcoidosis. Its safety and efficacy are supported by a retrospective study of 76 patients who had progressive sarcoidosis or had failed alternative immunosuppressive medications. A small, but significant improvement was seen in forced vital capacity (FVC) for pulmonary involvement, and a partial or good response was seen in 83% of the patients with extrapulmonary sarcoidosis (44). A steroid-sparing effect was also seen. The side effect and tolerability profiles were similar to studies in other rheumatologic diseases, with diarrhea and liver enzyme elevation being the most common side effects. Respiratory infections and neuropathy were the more serious, but less frequent side effects. A smaller study of 32 patients from 2004 also supported efficacy, with 78% of the patients showing improvement, including in patients who failed to respond to methotrexate (45). Leflunomide has similar lab monitoring requirements to methotrexate. Interestingly, both drugs have been associated with pneumonitis in rheumatoid arthritis, although leflunomide is less common than methotrexate for this confounding reaction (46).

Azathioprine, an inhibitor of purine metabolism, can also be considered as a steroid-sparing agent in patients with sarcoidosis, with similar efficacy compared to methotrexate to improve FVC and diffusing capacity (DLCO), and to taper corticosteroids (47). Azathioprine acts to decrease production circulating T cells and B cells, as well as increase apoptosis of circulating lymphocytes. Toxicity of azathioprine is affected by an individual’s thiopurine S-methyltransferase (TPMT) activity which plays a large role in drug metabolism. Genetic deficiency of this enzyme can increase the risk of myelosuppression and hepatotoxicity, although the role for routine testing prior to therapy is unclear given that most people who have myelosuppression do not have deficiency of TPMT (48, 49). The main side effects of azathioprine are gastrointestinal upset, myelosuppression, infections, and potential increased risk of malignancy (47). Side effects are comparable to methotrexate for use in sarcoidosis, although azathioprine did show a higher rate of infections (34.6% vs 18.1%, p=0.01) in one retrospective analysis (47). Doses of 2 mg per kilogram body weight per day were utilized in one study (50), whereas 100–150 mg per day in another (51).

Mycophenolate mofetil (MMF) is another second-line option that acts by inhibiting purine nucleotide synthesis specifically in lymphocytes and decreases production of autoantibodies by B cells; therefore, the drug is used commonly for immunosuppression in a variety of rheumatologic diseases and interstitial lung diseases (52). The largest series reported for use of MMF in sarcoidosis included 37 patients with primarily pulmonary sarcoidosis treated with MMF due to intolerance of an immunosuppressive regimen or treatment failure (53). A trend towards improved DLCO was seen in patients started on MMF, and a steroid-sparing effect was also noted. In a series of patients with neurosarcoidosis, 7 of 8 patients with central nervous system involvement showed remission of disease after 21 months of therapy (54). Three of four patients who had failed alternative immunosuppressive regimens appeared to respond to MMF. However, two patients with sarcoid myopathy did not appear to benefit from MMF. In all patients, no significant side effects were noted, concluding that this drug had a better tolerability profile than other immunosuppressive options and was effective for neurosarcoidosis with central involvement. Another small series of ten patients with chronic pulmonary sarcoidosis treated with MMF found similar steroid-sparing effect and little to no side effects, supporting its utility and tolerability in this patient population (55).

### Antimalarials

Chloroquine and hydroxychloroquine have been frequently used in treatment of sarcoidosis based on early randomized trials that showed a long-term benefit with chloroquine (56). However, based on the better safety profile, hydroxychloroquine is most often preferred. The mechanisms of action for hydroxychloroquine are varied; it can interfere with antigen presentation, prevent T cell activation, inhibit toll-like receptor signaling, and reduce inflammatory cytokines by T cells and B cells (57). Hydroxychloroquine has been particularly useful in cutaneous disease, hypercalcemia, and in some cases of neurosarcoidosis (58–60). Although gastrointestinal side effects are commonly reported with use of hydroxychloroquine, they are generally mild and well-tolerated (61).

### Inhaled Therapies

Inhaled corticosteroids have not been shown to have clear benefit in patients with sarcoidosis in a RCT (62), but given their low side effect profile and plausible mechanism of decreasing airway inflammation, they may play a role in maintenance, acute airway exacerbations, or cough (63, 64). However, given the lack of data and the likely small effects, consensus of sarcoidosis experts do not recommend inhaled corticosteroids for initial treatment of sarcoidosis (29). Another inhaled therapy, vasoactive intestinal peptide (VIP), has shown some potential effects; a single trial in 20 patients with sarcoidosis revealed that inhaled VIP decreased TNF-α production by alveolar macrophages and increased regulatory T cells in the lung. Reduced cough was seen in 75% of trial subjects, indicating its possible use for symptomatic relief (65). However, subsequent studies are needed to further dictate use of these inhaled therapies in clinical practice.

### Tumor Necrosis Factor (TNF) Antagonists and Biosimilars

TNF-α is a predominant cytokine, consistently elevated in active sarcoidosis as a product of macrophage activation, particularly at sites of granuloma formation (31). It is a major contributor to propagation of granulomatous inflammation. TNF-α by is elevated in progressive or steroid-resistant disease (66), and soluble TNF-α receptors are higher in BAL fluid of patients with active disease, signaling a role in pathogenesis (67). For this reason, the use of TNF inhibition has been studied as a therapeutic target. The use of TNF antagonists is supported by numerous compelling case reports and series in refractory sarcoidosis. Both infliximab and adalimumab are monoclonal antibodies targeted against TNF itself, with infliximab being chimeric and adalimumab being a humanized monoclonal antibody. In one double-blind, placebo-controlled Phase II trial of 138 patients with chronic pulmonary sarcoidosis, infliximab significantly increased the percent of predicted FVC compared to baseline by 2.5%, whereas placebo did not (68). The drug was not approved based on the perceived lack of clinical significance. Interestingly, however, post-hoc analysis suggested that patients with more severe disease and those with extrapulmonary disease may have derived the greatest benefit (68, 69). In addition, there were some improvements in reticular opacities on chest x-rays and decreases in inflammatory cytokines. A later examination of the data showed that infliximab had some benefit over placebo as measured by a novel extrapulmonary severity scoring tool, but the effect was not sustained after 24 weeks of therapy (70). However, a more recent long-term retrospective review of patients treated with infliximab for pulmonary and extrapulmonary sarcoidosis disputes this finding, as 58.5% of patients showed improvement of disease on pulmonary imaging up to 85 months of follow-up (71). Currently, infliximab is the most well-studied third-line agent and dosage recommendations are 3–5 mg/kg with maintenance therapy every 4–8 weeks after initial loading. The role of concurrent immunosuppression (e.g., methotrexate) to decrease formation of anti-drug antibodies for TNF inhibitors is unclear in sarcoidosis; its use is based on studies performed in rheumatoid arthritis and Crohn’s disease (72–74).

Increasing data for adalumimab would suggest a role for this TNF-α antagonist in sarcoidosis (75, 76). In one double-blind RCT of 16 patients with cutaneous sarcoidosis, adalimumab was associated with improved skin lesions (77). An open-label single-center study of 11 patients with refractory pulmonary sarcoidosis treated with 40 mg weekly of adalimumab showed that four patients had at least a 5% improvement in percent-predicted FVC (seven had stable FVC) and five had an improvement of at least 50 meters in 6-min walk distance (6MWD), with a total of eight who had improvement in one or the other (76). Another prospective observational study of ten patients with sarcoidosis refractory steroids and cytolytics, the PET avid activity seen with active inflammation was reduced in nine patients with the addition of adalumimab to the existing regimen, indicating responsive disease (78). In sarcoidosis patients with refractory posterior uveitis, adalumimab was associated with improvement in 85% of patients and stabilization in the remaining, supporting its use for ophthalmic sarcoidosis (79). Similarly, a recent series of 17 patients with refractory ocular sarcoidosis (predominately chronic relapsing panuveitis) showed efficacy of both adalimumab (40 mg subcutaneously every 2 weeks) and infliximab (5 mg per kg every 4–8 weeks) (80). The drugs were associated with an improvement in cells in the ocular anterior chamber, vitritis, macular thickness, and visual acuity, with a mean follow-up of 34 months. Corticosteroids were able to be tapered off in these cases. Adalimumab may also be effective for a proportion of patients who develop antibodies or resistance to infliximab therapy (81).

On the other hand, etanercept, a fusion protein that antagonizes the TNF receptor, has not shown clear efficacy in treatment of sarcoidosis. Therefore, this drug is not recommended for use in sarcoidosis. An open-label Phase II study in stages 2 and 3 pulmonary sarcoidosis was stopped early due to excessive treatment failures in patients treated with etanercept 25 mg subcutaneously twice weekly (82). Although five of seventeen patients appeared to respond to the drug, there were no clinical predictors of response that could be found, including TNF-α levels in the serum or BAL fluid. Similarly, in a double-blind randomized trial of 18 patients with refractory chronic ocular sarcoidosis, only three of the patients in the etanercept arm were able to decrease corticosteroid use, which was similar to the placebo group (83). This lack of efficacy may imply an inability for the drug to penetrate the vitreous cavity or may reflect the different mechanism of action by targeting the receptor, as compared to inhibition of the cytokine directly or lysing the cells that produce it, as infliximab does (83).

Similarly, ustekinumab and golimumab were tested in a three-arm double-blind RCT in chronic pulmonary sarcoidosis patients with a primary endpoint of FVC change (84). Golimumab is a fully human monoclonal IgG1 antibody specific for TNF-α, administered subcutaneously every month. Ustekinumab is an IL-12 and IL-23 inhibitor. After 16 weeks of therapy, no differences were observed between the intervention groups and placebo for lung function, although trends were seen in improvement of skin sarcoidosis. Interestingly, in this trial, the placebo group had improved lung function also, indicating that perhaps patient selection of spontaneously resolving patients could have biased results toward the null.

The advent of biosimilars has provided another, potentially less expensive, option for clinicians who desire an anti-TNF therapy. The biosimilar to infliximab has been tested in rheumatoid arthritis and found to have equivalent efficacy to its original form (PLANETRA trial) (85). A retrospective cohort study in 29 patients with sarcoidosis patients who received the infliximab biosimilar at a dose of 5 mg/kg/month showed improvement in FVC, health-related quality of life, reduction of standardized uptake value (SUV) on PET scans, and decreased sIL-2R biomarker (86). Another retrospective review of 20 neurosarcoidosis patients showed good efficacy and tolerable adverse events with infliximab biosimilar (87). Although promising, it will be important to follow patients closely who are treated with biosimilars; it is unclear if there are differences in immunogenicity that could complicate treatment dosages or switching of therapies (88).

### Anti-B Cell Therapy

Sarcoidosis has been associated with altered B cell homeostasis (89), high BAFF (11, 90), hypergammaglobulinemia, the presence of IgA-producing plasma cells near the granuloma (91), and autoimmune antibodies, suggesting that the humoral immunity may be playing a role. Consequently, it has been suggested that targeting of the B cells may influence the disease process (90). Rituximab, a chimeric monoclonal antibody against CD20+ B cells that reduce the mature circulating population, has been investigated in small studies (92–94). In one prospective phase I/II trial in ten patients with refractory pulmonary disease, five patients had a greater than 5% absolute improvement in FVC and five patients improved their 6MWD by 30 meters, with a total of seven patients having a response of one or the other (94). The results did not correlate with a patient’s pre-treatment immunoglobulin levels. Interestingly, two patients died of respiratory failure (thought to be due to progressive sarcoidosis) and there was one hospitalization for infectious pneumonia during the study follow-up. Lower et al. retrospectively assessed patients with ocular sarcoidosis (n=4) who were treated with rituximab and found that the therapy was effective as a steroid-sparing agent for three of the four, and well-tolerated except for neutropenia in two patients which resolved with lower doses and Staphylococcus aureus skin infections in another (95). Two of the sarcoidosis patients also had concurrent lung disease and incurred symptomatic pulmonary improvement. At this time, the role of rituximab as a third or fourth-line agent in sarcoidosis remains unclear, but future elucidation of the B cell actions in sarcoidosis will likely help in clarifying the use of this drug.

### Antifibrotics

Antifibrotics, now approved for treatment of idiopathic pulmonary fibrosis and progressive fibrotic interstitial lung diseases (ILDs), are also an enticing possibility for fibrotic sarcoidosis. The INBUILD trial, a positive RCT of nintadenib in various progressive fibrotic interstitial lung diseases included a few patients with fibrotic sarcoidosis (96). In the overall population of all patients, there was less decline in FVC (a difference of 107 ml) over 52 weeks as compared to placebo. The sarcoidosis patients were included in a group termed “other fibrosing ILDs” which included patients with sarcoidosis and exposure-related ILDs. This group made up approximately 12% of the study population, and therefore, the effects on purely sarcoidosis are not entirely clear. A trial of pirfenidone specifically for fibrotic sarcoidosis is also ongoing (NCT03260556). Future studies will be necessary to determine how antifibrotics may be incorporated into the management of sarcoidosis patients.

### Other Third-Line Agents

A few other immunosuppressive drugs have been reported as potential options in sarcoidosis, but lack a significant body of evidence in the form of RCTs or larger series. For example, cyclophosphamide has been reported as an effective treatment in corticosteroid-resistant disease in both neurosarcoidosis and cardiac sarcoidosis, and has been associated with a lower relapse rate for patients with neurosarcoidosis (97–99). However, with more responsive disease, the risk profile is less desirable than other steroid-sparing agents, making cyclophosphamide harder to justify for long-term treatment in milder disease. Adrenocorticotropic hormone analogue is also undergoing evaluation based on a multicenter RCT in chronic pulmonary sarcoidosis that showed improvement in lung function, imaging, and quality of life, combined with a steroid sparing effect (100). Although the drug holds historical FDA approval, there are little prior data to support its use in sarcoidosis; therefore, ongoing clinical trials will inform future use of this drug. Although less commonly used due to side effects and pill burden, pentoxifylline is an oral non-selective phosphodiesterase inhibitor that decreases cytokine production by suppression of macrophages. Its use is supported by one small RCT with 27 patients supporting a steroid-sparing effect, and one observational study in newly treated patients showing improvement or stability of disease with use of pentoxifylline (101, 102).

## Additional Therapies Under Investigation

Additional therapies targeting a variety of pathogenic mechanisms are also undergoing further study, but do not have enough evidence yet to be incorporated into treatment recommendations. For example, nicotine acts upon NFκB in macrophages to decrease cytokine production and acts to decrease the Th17/T-reg ratio by its effects on CD4+ lymphocytes, leading to evaluation of this compound as a treatment for sarcoidosis. A small RCT of 13 patients with sarcoidosis treated with transdermal nicotine versus standard treatment alone showed that those treated with nicotine had normalization of their TLR-2 and TLR-9 responsiveness and increased the T-reg response (103). Based on these preliminary data, a clinical trial is ensuing investigating the effect of nicotine on inflammatory biomarkers in patients with sarcoidosis (NCT02265874).

Another trial, entitled the CLEAR trial (Combined Levofloxacin, Ethambutol, Azithromycin and Rifampin), is targeting the hypothesis that mycobacteria are the elusive antigen in some cases of sarcoidosis. A pilot study of thirty patients with cutaneous sarcoidosis showed decrease in size of skin lesions and granulomatous burden with the CLEAR regimen (104). In a similar trial of 15 patients with chronic pulmonary sarcoidosis treated with CLEAR showed an improvement in walk distance, dyspnea (measured by St. George’s Respiratory Questionnaire), and FVC, although half of patients had to stop therapy due to intolerance (105). Results from a Phase II RCT in patients with progressive pulmonary sarcoidosis are awaited (NCT02024555).

Other drugs are being repurposed for treatment of sarcoidosis and are actively being investigated. For example, roflumilast, an oral anti-TNF agent approved for asthmatics with frequent exacerbations, is being evaluated for acute exacerbations of fibrotic sarcoidosis (NCT01830959). Apremilast, a phosphodiesterase-4 inhibitor approved for psoriasis that decreases production of TNF-α, interferon γ, IL-2, IL-12, and IL-23 was recently investigated in 15 patients with cutaneous sarcoidosis. In this study, skin lesions improved significantly after 12 weeks of therapy (106). A Phase 2a trial testing abatacept, a CTLA-4–Ig fusion protein that interferes with T-cell activation (currently approved for rheumatoid arthritis), is underway to understand safety and efficacy in chronic sarcoidosis (107). Similarly, a phase I/II trial using ATYR1923 (NCT03824392), a compound that downregulates T cell responses, cytokines and inflammatory fibrosis *via* modulation of neuropilin-2, is also ongoing in pulmonary sarcoidosis (108). Results from these studies may increase options for clinicians treating sarcoidosis.

Additionally, evolving research in granuloma formation and propagation has suggested new potential therapeutic targets. For example, mTORC1 activation of macrophages has been associated with disease progression, and alveolar macrophages from sarcoidosis patients have upregulation of interleukin-1 receptor associated kinases (IRAK1 and IRAK-M) and receptor interacting protein 2 (Rip2) (109, 110). Interference with these pathways may result in decrease of granuloma formation.

The role of vitamin D in prevention or treatment of sarcoidosis has also been debated, based on the potential immunomodulatory role in Th1 inflammation. Vitamin D has been shown *in vitro* to inhibit proliferation of Th1 lymphocytes, as well as suppress antigen presentation and activation of macrophages, all of which could potentially diminish granuloma formation and propagation (111, 112). Vitamin D may also modulate dendritic cell differentiation, decreasing the antigen presenting capabilities of the immune response (113). Clinically, low serum levels of 25-hydroxyvitaminD have been associated with active disease (114). However, treatment of sarcoidosis is complicated by the dysregulated calcium metabolism that occurs with granulomatous inflammation. The macrophage converts vitamin D to its active form, 1,25-dihydroxyvitaminD, *via* 1-alpha-hydroxylase, which is activated in sarcoidosis irregardless of the parathyroid feedback mechanisms which normally control calcium levels. Thereby, up to 10% of patients with sarcoidosis will have hypercalcemia and even a higher percentage will have hypercalciuria, making vitamin D supplementation increasingly risky if not closely monitored. A small clinical trial of 16 sarcoidosis subjects with normal serum ionized calcium levels and vitamin D deficiency showed that treatment with ergocalciferol increased the storage form of vitamin D, whereas decreased the active form (1,25-dihydroxyvitaminD) and angiotensin converting enzyme levels, suggesting an effect on granulomatous inflammation (115). Asymptomatic increases in serum calcium levels were seen in three of the patients in this trial. Larger clinical trials will be necessary for future recommendations regarding vitamin D as a treatment modality regarding both safety and efficacy.

## Choosing a Treatment Option

Deciding on the most appropriate treatment regimen for a patient is often a complex interplay of disease characteristics (such as immediate risk of severe organ damage), familiarity of therapies by the clinician, side effect profiles, and patient preferences. The choice can be influenced by a patient’s age, alcohol intake, likelihood of pregnancy, concurrent medications, or comorbidities such as diabetes, liver or kidney dysfunction. Given the polypharmacy often involved in the patient regimen, drug interactions should be considered in medication choice. Corticosteroids are most often first line therapy for any type of sarcoidosis, but second or third-line therapies can be considered in cases where corticosteroids are risky (decompensated heart failure, uncontrolled diabetes, severe obesity, uncontrolled hypertension, glaucoma) or these drugs can be added in the more aggressive treatment of life-threatening or severe organ derangement such as can be seen in neurosarcoidosis, ophthalmic injury, or severe infiltrative heart disease. On a more chronic, outpatient regimen, steroid-sparing agents are often used when corticosteroids cannot be reduced to reasonable doses (less than 10–15 mg per day), there is corticosteroid toxicity, or the anticipated course of treatment is lengthy.

### Length of Treatment

Duration of therapy, whether with corticosteroids or other immunosuppressive treatment, is generally considered to be approximately 1 year, based on data suggesting an increased risk of relapse with shorter courses (23, 116–118). The British Thoracic Society Sarcoidosis Study found that in patients with asymptomatic, but radiographically evident pulmonary sarcoidosis, who were treated empirically with long-term therapy of 18 months versus “selective” therapy based on development of symptoms or radiographic progression, the patients who received long-term therapy had greater improvements in symptoms, respiratory function, and radiographic appearances than those in the selectively treated group (119). At this point, there are no sensitive or specific biomarkers to predict relapse, leading to blanket generalization of longer duration (120); however, shorter tapers can be considered based on symptoms and overall response to therapy on an individual basis. This is supported by recent data from a prospective study of 21 patients with pulmonary sarcoidosis showing that most of the improvement in pulmonary function (measured by home spirometry), was seen in the first month after initiation of treatment (121). A smaller additional improvement was seen by three months, indicating that steroids may be able to be tapered within the first three months to a tolerable, long-term dose that would lessen the toxicity for patients. Additionally, this study showed that the improvement in fatigue as measured by the Fatigue Assessment Scale (FAS) and dyspnea as measured by the Medical Research Council dyspnea scale also occurred within the first month of treatment (121).

### Organ-Specific Therapy

The question of whether there are organ systems which benefit from a particular steroid sparing agent is not wholly clear, and generally, extrapulmonary sarcoidosis is treated with the same algorithms as pulmonary sarcoidosis (28). However, some extrapolations from prior study have suggested preferences for certain therapies depending on sarcoidosis phenotype. For example, hydroxychloroquine has been suggested as quite effective for cutaneous sarcoidosis, but not considered highly effective for pulmonary disease (60). Hydroxychloroquine also impairs 25(OH)D3-1-α-hydroxylase, and therefore, it is often chosen to treat hypercalcemia (59). Similarly, leflunomide seemed to be more effective for lung, skin, eye, and sinus disease, and less so for neurosarcoidosis and musculoskeletal disease in one retrospective study (44). In another series, MMF was quite effective for neurosarcoidosis, resulting in some favoritism of this drug for treatment of neurosarcoidosis (54). However, a more recent review of long-term (median follow-up 8 years) outcomes of a large series of 234 patients from France, found that a lower risk of neurologic relapse was seen in patients treated cyclophosphamide (HR 0.26, 95%CI 0.11–0.59), methotrexate (HR 0.47; 95%CI 0.25–0.87), and hydroxychloroquine (HR 0.37, 95% CI 0.15–0.92) (97). This effect was also seen in risk of overall relapse rate, in addition to neurologic relapse. On the other hand, mycophenolate and azathioprine were not associated with a decreased relapse rate, either overall or specifically in the neurologic system. Infliximab was associated with decreased overall relapse rate (neurologic or other organ), and a trend was seen for decrease in neurologic relapses (HR 0.16, CI 0.021–1.24, p=0.08) Conversely, glucocorticoids alone were associated with a decrease in any organ relapse rate, but not specifically for neurologic relapse, perhaps suggesting a need for dual therapy in these cases. In current management, there are few comparative effectiveness studies that can dictate choice of agent based on organ involvement, and therefore, decisions are often made on tolerability, response, and physician experience.

### Predictors of Clinical Course and Treatment Response

Although some demographic and clinical characteristics have been associated with worse outcomes and increased disease severity in the broader sarcoidosis population, there are no patient-specific biomarkers used in clinical practice that can directly predict who will progress and necessitate treatment, nor are there clear biomarkers that will predict relapse in any one individual patient. However, increasing data shows promise for potential biomarkers. Multiple differing HLA haplotypes seem to track with both disease onset and differing presentations, implying that the genetic profile of the immune system can account for some of the varied clinical manifestations (122). For instance, the HLA-A1 and HLA-B8 have been associated with acute onset of sarcoidosis, and HLA-DR14(6) and DR15(2) are associated with chronic disease (123, 124). With further insights into abnormal T-regulatory function, recent data has shown an increased proportion of ‘exhausted’ T-reg cells is associated with chronic sarcoidosis (125). Conversely, the presence of high levels of functioning T-regs in tissues are associated with more self-limited disease (126). The presence of B cells and aberrant proportions of B cells may also signal poorer outcomes. BAFF has also correlated with multi-system disease, and low NFKβ p65 protein on T cells and B cells has been associated with increased severity of disease (90, 127, 128).

For treatment response, genetic polymorphisms may account for some of the variability in treatment response by therapy. For example, a study of 111 refractory sarcoidosis patients who were started on infliximab or adalimumab showed that patients without the TNF-alpha-308A variant allele (GG genotype) had a three -fold higher response to the TNF antagonists compared to those with the allele (129). Higher soluble TNF-receptor-2 expression levels also seem to correlate with response to infliximab (130). Additionally, a small study of five patients with CD4+ lymphopenia showed both clinical improvement in and an increase in CD4+ T cell counts, suggesting that this phenotype may be particularly responsive to infliximab (131). Increasing research and understanding of the intricate mechanisms of pathophysiology may yield future personalized prognostic markers.

## Treatment-Associated Issues

Although immunosuppression dominates the forefront of sarcoidosis therapy, in reality, holistic treatment of patients with sarcoidosis often involves addressing the comorbid conditions associated with the disease and mitigating side effects of immunosuppression. Sarcoidosis can cause a number of “danger situations”, including fibrocystic sarcoidosis, sarcoidosis-associated pulmonary hypertension (SAPH), bronchiectasis, and mycetomas (132). Additionally, “parasarcoidosis” syndromes are associated with the disease, including small fiber neuropathy, cognitive dysfunction, chronic pain, and fatigue; each of these issues can cause significant disability and burden upon patients, and often, do not respond desirably to traditional immunosuppressive therapies (25). Last, the immunosuppressive treatments themselves contribute to predictable comorbidities, including infections, fatigue, and malaise. Corticosteroids are highly associated with obesity, malaise, decreased bone density, cataracts, hyperglycemia and edema (133, 134). Additionally, each steroid-sparing agent can be associated with organ toxicities in the liver, kidney, or bone marrow. One cross-sectional study assessing gastrointestinal (GI) side effects in patients from the United States, United Kingdom, and the Netherlands found that the most important GI side effect was weight gain related to corticosteroid use; methotrexate was associated with nausea and diarrhea. Vomiting and weight loss were most associated with azathioprine and mycophenolate (61). Last, paradoxically, some of the drugs used to treat sarcoidosis (e.g., the TNF-antagonists) have been associated with development of a sarcoid-like reaction (135).

Strategies to circumvent detrimental toxicities of both corticosteroids and alternative immunosuppression are important to incorporate into the multidisciplinary management of patients. For example, maintaining routine recommended vaccinations for infections such as influenza or pneumococcus should be considered (136, 137). Additionally, bone health should be addressed upon initiation of corticosteroids and regularly thereafter; clinical risk assessment (yearly) and intermittent bone mineral density testing can be incorporated based on duration of corticosteroid use, age, and risk scores (138). Treatment often includes bisphosphonates or other anti-fracture medications. Bone health management can be somewhat complicated given the need for calcium and vitamin D supplementation, but can be done safely with close monitoring. Similarly, patients should adhere to recommended laboratory monitoring intervals specific to their immunosuppressive medication to avoid irreversible organ injury (139). Regular eye exams can be helpful to evaluate for glaucoma or cataracts that can result from corticosteroid use.

### Cavitary Lung Disease and Bronchiectasis

Fibrotic sarcoidosis has been historically difficult to treat given lack of treatment options and resultant complications of fibrosis (140). Pulmonary cavities can occur in patients with sarcoidosis related to progressive fibrosis of the lung (141). Mycetomas, caused by *Aspergillus* within lung cavities, can cause hemoptysis or lead to invasive infection. Similarly, bronchiectasis resultant of fibrotic sarcoidosis can also complicate treatment. In these cases, use of antibiotics and pulmonary therapies including bronchodilators, mucolytics, and chest physiotherapy is often most effective (140). Immunosuppression may be counterproductive in these cases, exacerbating chronic or repeated infection.

### Pulmonary Hypertension

Pulmonary arterial hypertension can be a complication of sarcoidosis. It is associated with increased mortality, especially among those individuals awaiting lung transplantation (142, 143). Pulmonary hypertension in sarcoidosis may be due to granulomatous vasculitis, pulmonary artery compression by lymphadenopathy, left heart dysfunction due to myocardial involvement, porto-pulmonary hypertension in those with associated liver disease, parenchymal and vascular destruction due to fibrosis, or hypoxic vasoconstriction related to parenchymal abnormalities. Additionally, the associated risk of thromboembolic disease can lead to pulmonary emboli (and subsequent pulmonary hypertension), requiring a high index of suspicion (144). Pulmonary hypertension should be suspected if there is a drop in functional status or DLCO in cases of stable pulmonary parenchymal disease. Upon CT evaluation, pulmonary artery diameter indexed to body surface area correlates with the presence of sarcoidosis-associated pulmonary hypertension (SAPH) and may raise also suspicion (145).

Because of the varied contributing pathophysiology (some of which may be overlapping), the treatment of pulmonary hypertension in sarcoidosis can be complex. Corticosteroid treatment may result in the improvement of pulmonary pressures in some, but not all cases, reflecting the differing mechanisms of pulmonary hypertension. Case series suggest benefit of prostacyclin analogues in some patients and endothelin receptor antagonists (ERAs) have conflicting data reports (146–148). Retrospective series have shown that ERAs may decrease pulmonary pressures and improve functional and exercise capacity in some individuals (149). However, a RCT of 35 patients randomized to bosentan 125 mg twice daily versus placebo failed to show a functional improvement in walk distance, although this study did show small, but statistically significant, improvement in mean pulmonary artery pressure and pulmonary vascular resistance (PVR) (150). Two of the treated patients had increased need for oxygen, possibly indicating a deleterious effect on the compensatory mechanisms of hypoxic vasoconstriction seen in patients with concomitant parenchymal lung disease. Another study, an open-label proof of concept trial of 21 SAPH patients treated with ambrisentan, showed that, in the patients who completed therapy over 24 weeks, there was a non-statistical improvement in walk distance and dyspnea as measured by the St. Georges Respiratory Questionnaire (146). In this trial, drop-out rate was 52% primarily driven by intolerance of the drug. A more recent retrospective review of the French Pulmonary Hypertension Registry between 2004–2015 showed that patients on pulmonary vasodilator therapy (including phosphodiesterase-5 inhibitors, prostacyclin analogues, and ERAs) appeared to have hemodynamic benefit, but lacked a functional benefit from PAH therapies, similar to results from the bosentan RCT (142). Clinical trials with selexipag (a non-prostanoid prostacyclin receptor agonist) and inhaled treprostinil in patients with SAPH is have recently been initiated (NCT03942211, NCT03814317) with the primary outcome of hemodynamic measurements, including PVR. Given the multiple potential causes of pulmonary hypertension in this population, current optimal management for SAPH is unclear. Vasodilator treatments should be used with caution and careful patient selection is advised.

### Lung Transplantation

In case of progressive organ failure, transplantation can be considered in patients with sarcoidosis. Approximately 3% of lung transplants done in the United States are due to sarcoidosis (151). Successful outcomes have been reported for lung, heart, and liver disease, comparable to transplantation for alternative causes (151, 152). Interestingly, despite post-transplant immunosuppression, recurrence of non-necrotizing granulomas in the transplanted organ is common, and thought to be derived from the recipient (153, 154). One analysis of DNA from transbronchial biopsies of lung transplant recipients with recurrent granulomatous inflammation found increased percentage of recipient DNA in the epitheloid clusters suggesting repopulation by host macrophages (154). Granulomas can be found on routine transbronchial biopsies in follow-up and often resolve spontaneously (155). Generally, the presence of granulomatous inflammation does not seem to affect overall survival in most patients (151, 156).

### Small Fiber Neuropathy, Cognitive Dysfunction, and Fatigue

Small Fiber Neuropathy in sarcoidosis causes chronic pain, autonomic dysfunction, and altered sensation (157). Treatment for small-fiber neuropathy has been notoriously difficult, with failure to respond to most traditional therapies such as corticosteroids, MTX, AZA, MMF, and therefore, symptomatic treatment for neuropathic pain is usually considered (158). Additionally, the anti-TNF agents, both infliximab and adalumimab, have some suggestion of improving sarcoidosis-related fatigue and cognitive difficulties, as compared to other immunosuppressive agents (159). Intravenous immunoglobulin has also been suggested and has shown efficacy in a proportion of patients (160). ARA 290, a peptide that targets the innate repair receptor to decrease cytokine production and tissue inflammation, is also currently under study given supporting preliminary data in a small RCT of 22 patients showing a reduction in small fiber neuropathy symptoms (including pain) when treated with 28 days of ARA 290 (161).

Fatigue in sarcoidosis is extremely common. Its presence is an interplay of inflammation, musculoskeletal disease, mental health, treatment side effects, and sleep issues (162, 163). Given the association of sarcoidosis and sleep apnea, sleep evaluation is recommended in the workup of fatigue in this population (164). Both armodafinil and dexmethylphenidate have shown some benefit for sarcoid-related fatigue in very small studies of 15 patients or less when added to their immunosuppressive regimen (165, 166). Given the above potential benefits seen with the TNF antagonists, further study with these drugs should include fatigue as an endpoint. Treatment of depression and anxiety, both common confounding ailments, should also be evaluated and considered (167).

### Physical Training/Pulmonary Rehabilitation

Physical training studies in sarcoidosis have suggested that a structured physical activity program can improve exercise capacity, muscle strength, and fatigue in sarcoidosis (168, 169). Additionally, physical training regimens appear to improve overall physical and psychological well-being, suggesting a role in the treatment of sarcoidosis. Pulmonary rehabilitation and training have been incorporated into expert consensus recommendations (170).

## Conclusions

Treatment of sarcoidosis is complex and non-standardized for clinicians and patients. Further research is necessary to inform clinical guidelines and provide higher quality evidence for treatment regimens. Drug development is challenging due to the lack of animal model and rudimentary understanding of pathogenesis. Additionally, research should involve clinical trial design and prognostic biomarkers to appropriately select and evaluate those patients who will require therapy. Advancing the knowledge regarding etiology and pathophysiology may one day lead to prevention or cure. Future treatments will likely involve prevention of exposure, treatment of an antigen, mitigation of granulomatous inflammation, and interrupting fibrotic pathways. Management decisions should evolve to include personalized medicine based on pharmacogenomics and sarcoidosis phenotype, as well as patient-centered approaches to incorporate immunosuppression, symptom control, and treatment of comorbid conditions.

## Author Contributions

The author confirms being the sole contributor of this work and has approved it for publication.

## Funding

AG is funded, in part, by the Federal Drug Administration Grant#FD-R-0005993 to study the natural history of sarcoidosis.

## Conflict of Interest

The author declares that the research was conducted in the absence of any commercial or financial relationships that could be construed as a potential conflict of interest.
